# Supplementary figures and images for: Human Inferences about Sequences: A Minimal Transition Probability Model
Source: PLoS Comput Biol. 2016 Dec 28;12(12):e1005260. doi: 10.1371/journal.pcbi.1005260 (PMC5193331; doi:10.1371/journal.pcbi.1005260)

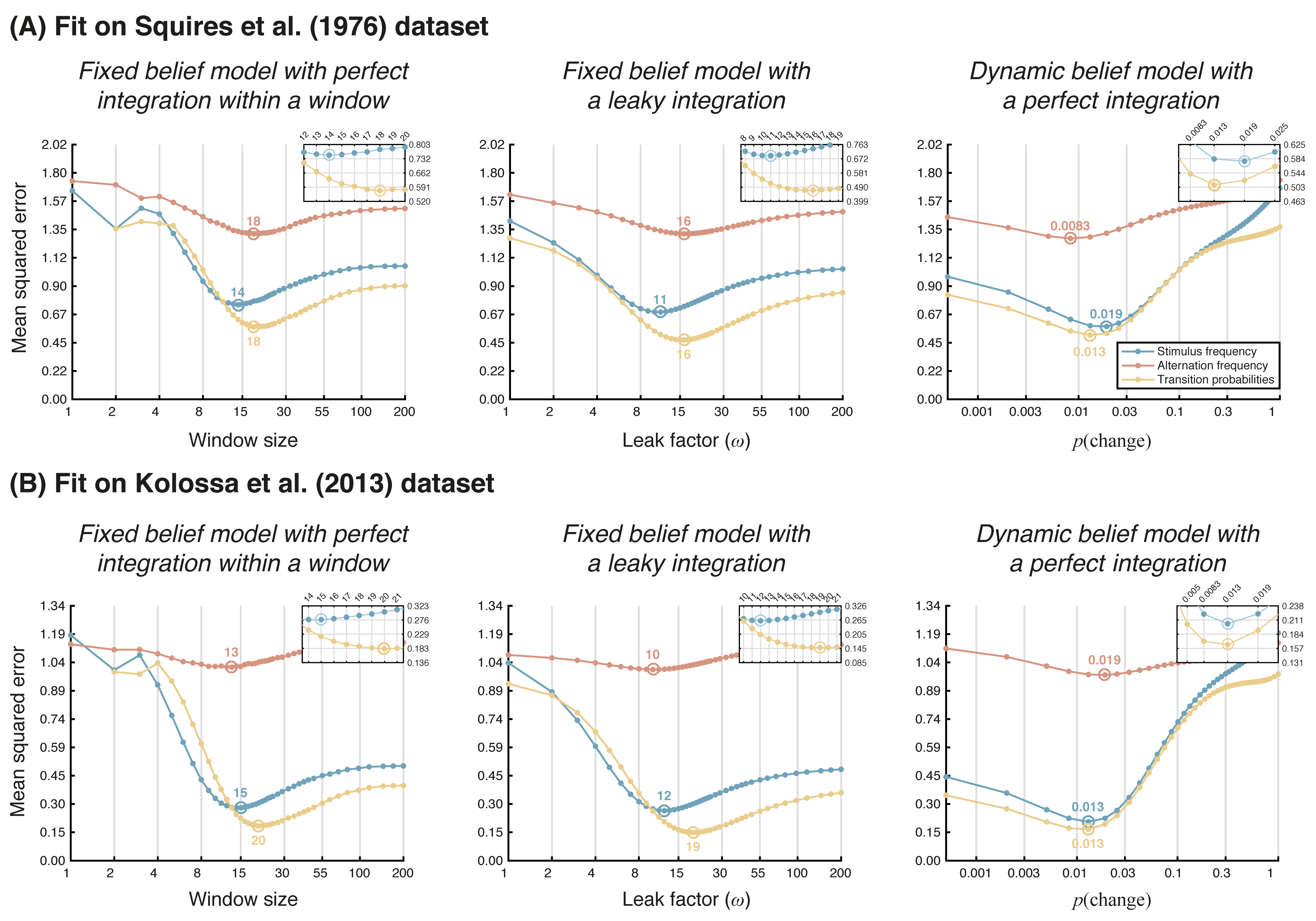

Supplement: S1 Fig — The plot shows the mean squared error of model fit, for different models, different values of their free parameter and different datasets. The inset shows a zoom around the best-fitting parameter. Note that different models have different inference styles: fixed belief with perfect integration within a window of observation, fixed belief with leaky integration and dynamic belief (presented in different columns) and they estimate different statistics: item frequency, alternation frequency and transition probabilities (presented as colored lines within each plot). (TIF) [file pcbi.1005260.s001.tif]

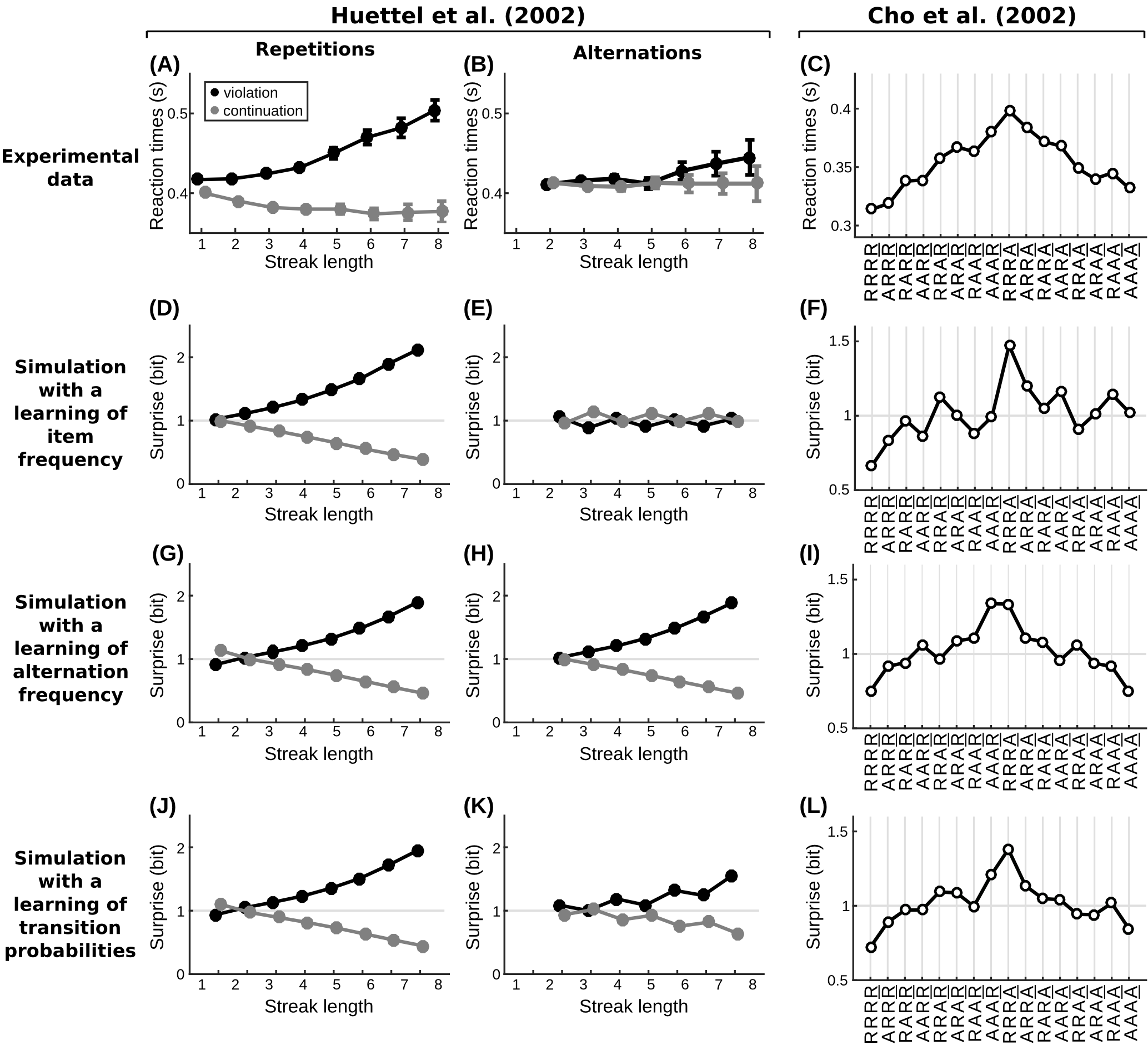

Supplement: S2 Fig — This figure is similar to Fig 4. The only difference is that theoretical surprise levels are computed from the dynamic belief model (see Methods). The free parameter of the models, the a priori change probability for the estimated statistics, was selected independently, as the best fitting value for Squires et al. data. (TIF) [file pcbi.1005260.s002.tif]

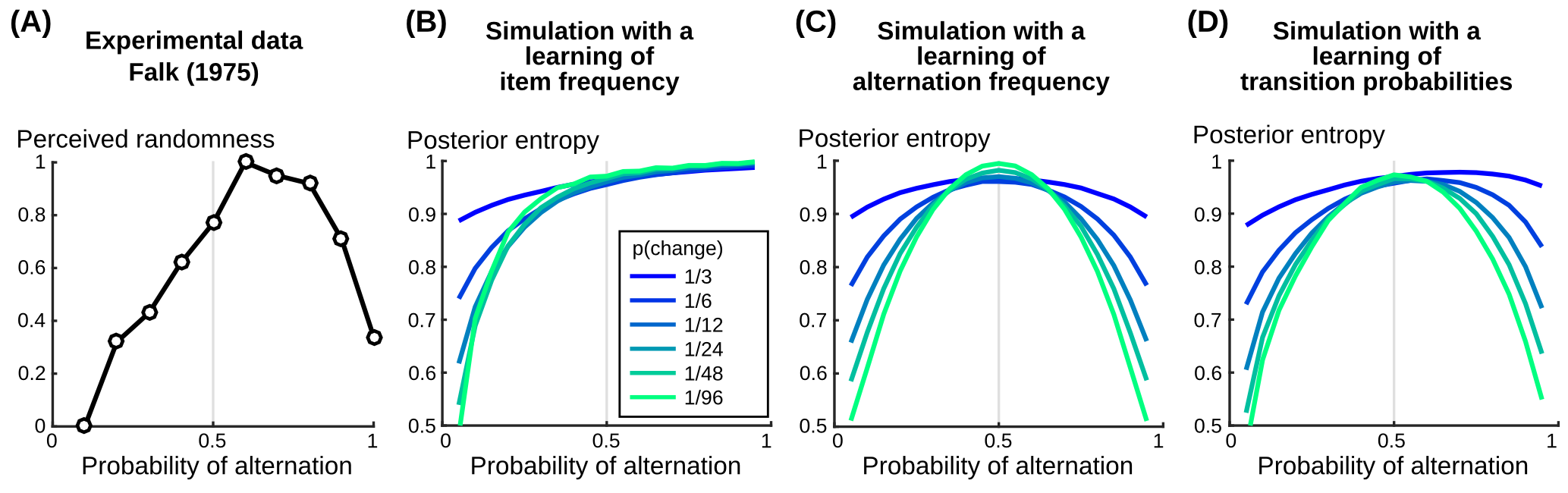

Supplement: S3 Fig — This figure is similar to Fig 5. The only difference is that theoretical entropy levels are computed from the dynamic belief model (see Methods) using different a priori change probabilities for the estimated statistics. (TIF) [file pcbi.1005260.s003.tif]

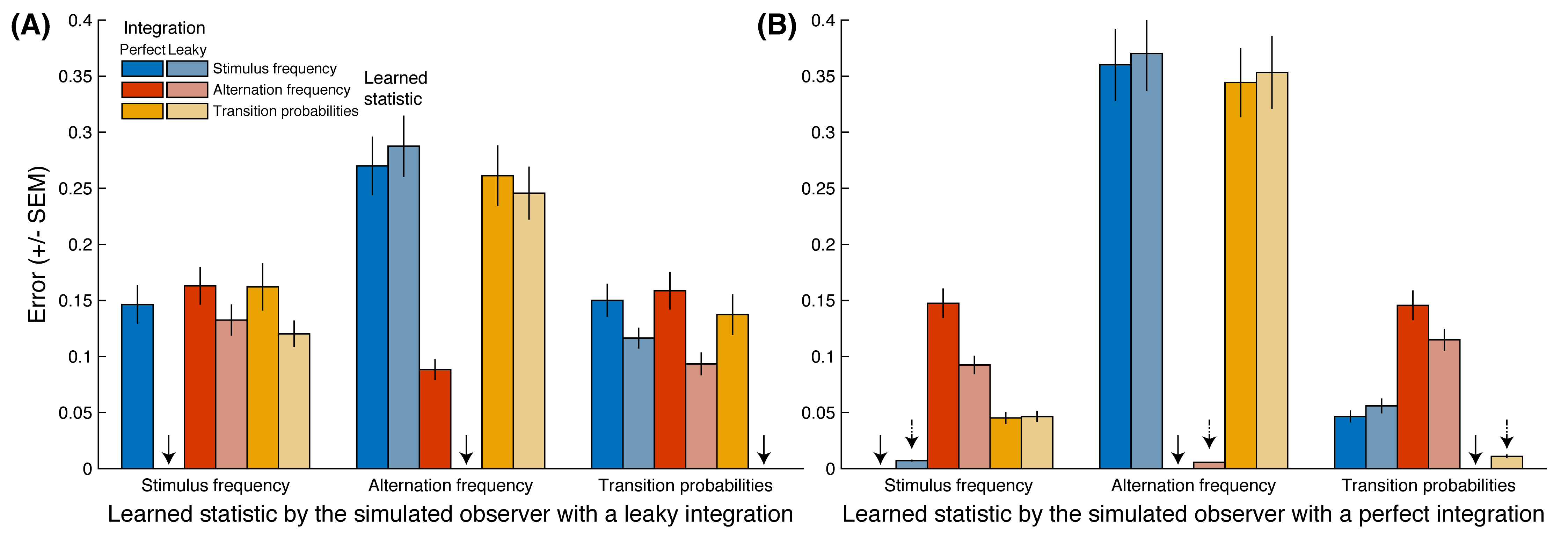

Supplement: S4 Fig — We estimated whether the models we consider make predictions that can be distinguished quantitatively from one another. We started by simulating the results of the Squires et al. experiment, using the best-fitting parameters of each model. As in Squires et al., each simulated data set contained 48 data points, corresponding to 16 patterns times 3 block types. We then estimated the ability of a given model to recover the predictions of another model with a leave-one out procedure. More precisely, we took the 48 simulated values of a given model and we adjusted the free parameters of another model to 47 of these simulated values, leaving one out. Given these fitted parameters, we then computed the prediction of the second model about the left-out point and we measured the error (the unsigned difference) compared with the original simulation. The bars show, for all pairs of models, the mean error and SEM across left-out points. The comparison of a model against itself yields no error (see arrows). Different models that make similar predictions should yield an error close to 0. This occurs in only one case (see dashed arrows): the predictions of a model learning perfectly a given statistic can be recovered almost exactly by a model learning the same statistic with a leaky integration. This is because the leak can be adjusted so as to approach a perfect integration. Critically, models that learn different statistics all produced quantitatively different predictions. (TIF) [file pcbi.1005260.s004.tif]

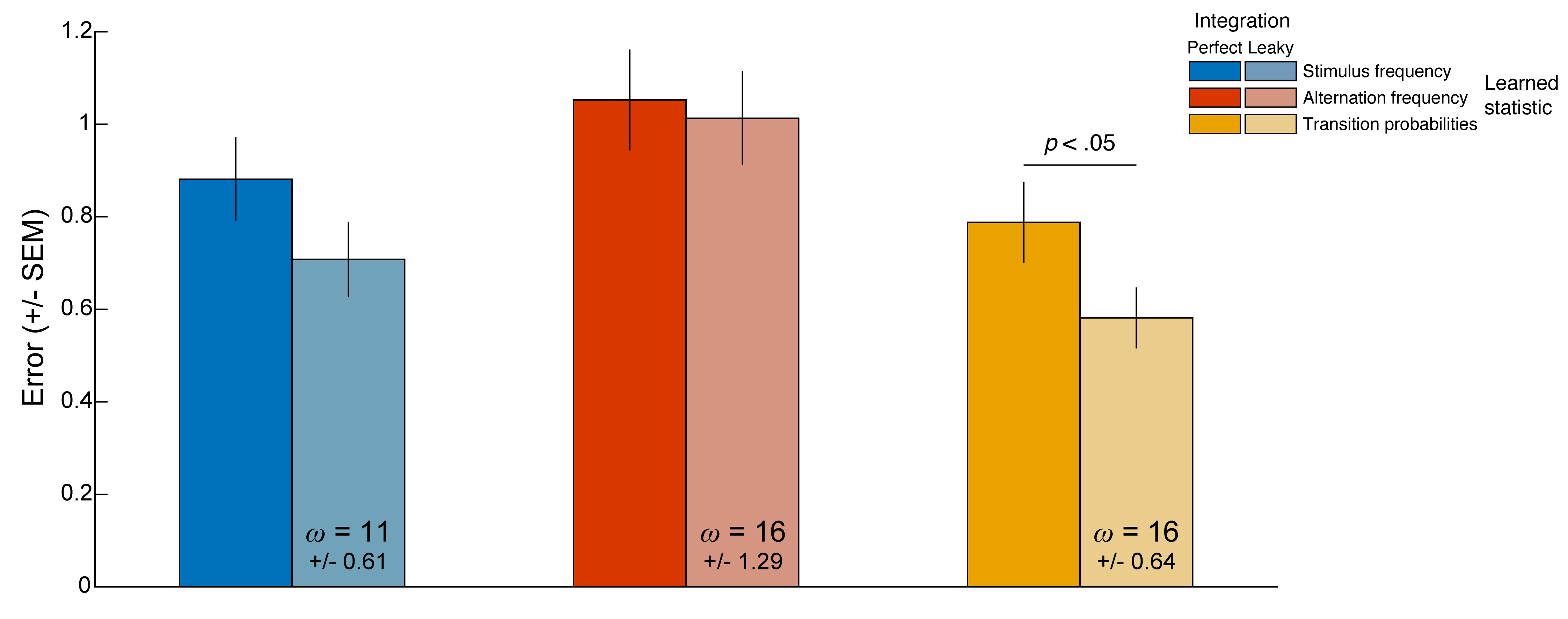

Supplement: S5 Fig — We estimated the predictive accuracy of each model using a leave-one out procedure. The parameters of each model (the offset and slope of the linear transformation from theoretical surprise to P300 data, and the leak of leaky integration models) were fitted to all data points but one. We then measured the error of a given model, as the distance between the actual left-out point and the value predicted by the model and its fitted parameters. Since Squires et al. report data for 16 patterns in 3 different block types, we repeated the leave-one out procedure 16 * 3 = 48 times. Bars show the mean error with SEM, and the numbers indicate the mean (and SEM) of the best-fitting leak parameter ω across left-out points. The model learning local estimates of transition probabilities achieved the best predictive accuracy, i.e. the smallest error. (TIF) [file pcbi.1005260.s005.tif]
