# Supplementary material for: Human Inferences about Sequences: A Minimal Transition Probability Model
Source: PLoS Comput Biol. 2016 Dec 28;12(12):e1005260. doi: 10.1371/journal.pcbi.1005260 (PMC5193331; doi:10.1371/journal.pcbi.1005260)
Supplement: S1 Text — (DOC) [file pcbi.1005260.s006.doc]

# Supplementary equations

Several statistics are embedded in the space of transition probabilities. Indeed, transition probabilities fully specify the frequency of items:

$p\left(X\right)=\frac{p\langle(X|Y)\rangle}{p\langle(X|Y\rangle)+p\langle(Y|X)\rangle}$ Eq. S1

They also fully specify the frequency of ordered pairs of items:

$\begin{matrix} p\left(X,X\right)=p\langle(X|X\rangle)p\left(X\right) \\ =p\langle(X|X\rangle)\frac{p\langle(X|Y\rangle)}{p\langle(X|Y\rangle)+p\langle(Y|X\rangle)} \\ \\ p\left(X,Y\right)=p\langle(Y|X\rangle)p\left(X\right) \\ =p\langle(Y|X\rangle)\frac{p\langle(X|Y\rangle)}{p\langle(X|Y\rangle)+p\langle(Y|X\rangle)} \\ \\ p\left(Y,X\right)=p\langle(X|Y\rangle)p\left(Y\right) \\ =p\langle(X|Y\rangle)\frac{p(\langle Y|X\rangle)}{p\langle(Y|X\rangle)+p\langle(X|Y\rangle)} \\ \\ p\left(Y,Y\right)=p\langle(Y|Y\rangle)p\left(Y\right) \\ =p\langle(Y|Y\rangle)\frac{p\langle(Y|X\rangle)}{p\langle(X|Y\rangle)+p\langle(Y|X\rangle)} \end{matrix}$ Eq. S2

And they fully specify the frequency of alternations:

$\begin{matrix} p\left(A\right)=p\left(X,Y\right)+p\left(Y,X\right) \\ p\left(R\right)=2\frac{p\langle(Y|X)\rangle p\langle(X|Y)\rangle}{p\langle(X|Y\rangle)+p\langle(Y|X\rangle)} \end{matrix}$ Eq. S3
